# Blast Exposure Induces Acute Alterations in Circadian Clock Genes in the Hypothalamus and Pineal Gland in Rats: An Exploratory Study

**DOI:** 10.1089/neur.2023.0048

**Published:** 2023-08-31

**Authors:** Manoj Govindarajulu, Mital Y. Patel, Jishnu Krishnan, Joseph B. Long, Peethambaran Arun

**Affiliations:** Blast-Induced Neurotrauma Branch, Center for Military Psychiatry and Neuroscience, Walter Reed Army Institute of Research, Silver Spring, Maryland, USA.

**Keywords:** blast injury, circadian rhythm, clock genes, hypocretin, hypothalamus, pineal gland

## Abstract

Blast-induced traumatic brain injury (bTBI) frequently results in sleep and circadian rhythm disturbances. We have investigated whether dysregulation of circadian rhythm after bTBI is mediated by dysregulation of clock genes in the hypothalamus and pineal gland of rats at acute (24 h) and chronic (1 month) time points post-blast. Expression of core circadian genes (*Bmal1*, *Clock*, *Per1*, *Per2*, *Cry1*, and *Cry2*) in the hypothalamus and pineal gland were quantified using quantitative real-time polymerase chain reaction. Hypocretin (*Hcrt*) and hypocretin receptor (*Hcrtr1* and *Hcrtr2*) expression in the hypothalamus were also quantified along with plasma corticosterone levels. Blast-exposed rats showed a statistically significant increase in *Bmal1* and decreases in *Per1*, *Per2*, and *Cry2* in the pineal gland at 24 h post-blast in rats euthanized at night. In the hypothalamus, increases in *Bmal1*, *Cry1*, and *Cry2* were noted along with decreases in *Per1* and *Per2* gene expression at 24 h post-blast in rats euthanized at night. Except for *Bmal1* in the hypothalamus, no statistically significant changes in expression of any of the clock genes were detected in the hypothalamus or pineal gland samples collected during daylight post-blast. In the hypothalamus, a decrease in *Hcrt* associated with increases in *Hcrtr1* and *Hcrtr2* were noted at 24 h post-blast in rats euthanized during daytime and nighttime. Increased plasma corticosterone was noted at 24 h post-blast in samples collected at night. No statistically significant changes in any of the core circadian genes—hypocretin, hypocretin receptors, or plasma corticosterone—were observed in the samples collected at 1 month post-blast injury. Blast exposure causes differential expression of core circadian genes in the hypothalamus and pineal gland during nighttime, along with dysregulation of hypocretin and its receptors, which might play a key role in the sleep disruptions associated with bTBI.

## Introduction

Traumatic brain injury (TBI) is a major medical concern, and because of increasing global terrorism, there is increased prevalence of blast-induced TBI (bTBI) among U.S. military service members, veterans, and civilians.^1,2^ Among U.S. service members, there have been ∼450,000 brain injuries sustained since 2000. Interestingly, blast-related TBIs made up a significant portion of these injuries, leading to several long-term neurological deficits.^3^ One of the most predominant, debilitating, and persistent sequelae of TBI include sleep-wake disturbances.^4–6^ A meta-analysis involving human TBI found that sleep-wake disturbances post-TBI occurred in ∼50% of persons.^7^ Interestingly, self-report rates of sleep-wake disturbances post-TBI are significantly higher in military populations even after a mild TBI.^8^ Identification and treatment of sleep-wake disturbances in TBI patients are important and can complement other efforts to promote maximum functional recovery.

Sleep and wakefulness are controlled by circadian rhythms, which are internally driven cycles that rise and fall in a 24-h period. The mammalian primary circadian clock is controlled by the suprachiasmatic nucleus (SCN), located in the hypothalamus, and the SCN helps coordinate these rhythms throughout the body. Studies indicate that bilateral lesions of the SCN result in loss of nocturnal and circadian rhythms.^9^ The molecular signaling pathways governing the circadian rhythms consist of the canonical clock gene feedback loops consisting of core circadian clock genes (*Bmal1*, *Clock*, *Per1*, *Per2*, *Cry1*, and *Cry2*). The major loop involves helix-loop-helix Per-ARNT-SIM domain-containing transcription factors CLOCK and BMAL1, which form a complex and regulate the transcription of other clock genes, *Periods* (*Per1* and *Per2*) and *Cryptochromes* (*Cry1* and *Cry2*), the products of which in turn inhibit CLOCK/BMAL1 transcriptional activity and their own expression, thus forming a negative feedback mechanism.^10^

These genes are expressed in different regions of the brain and other peripheral organs and subserve several vital functions, including regulation of circadian rhythms both in the brain and the periphery. Melatonin, a hormone important for regulating sleep, is secreted by the pineal gland in a circadian manner as influenced by the SCN. Further, studies indicate that melatonin in turn acts on the SCN and directly influences the circadian clock mechanisms, indicating that a complex interaction between the SCN and pineal gland plays a crucial role in the regulation of sleep.^11^

In addition to the core clock genes, hypothalamic neuropeptides such as orexins/hypocretins play a crucial role in sleep-wake behavior. Hypocretin (*Hcrt*) is essential for promoting wakefulness and stabilizing the wakefulness state.^12^ Activity of the orexin neurons is regulated by the circadian stimulus and is increased during the wake phase, which correlates with heightened motor activity in nocturnal rodents.^13,14^ Further, the rhythmic expression of orexin receptors has been linked to changes in clock gene expression. Specifically, orexin receptor type 2 shows a strong and significant correlation with the *Bmal1* gene in the hypothalamus.^15^ In a non-blast-related TBI model in mice, upregulation of hypocretin receptor 1 (*Hcrtr1*) was noted beginning at 6 h after TBI and peaking at 1 day post-injury.^16^

Only limited studies have investigated the way circadian rhythm disruption after TBI might be mediated by changes in expression of clock genes in the SCN and other brain regions. In a fluid-percussion model of TBI in rats, increased expression of *Cry1* and *Bmal1* genes in the SCN and decreased expression of *Cry1, Bmal1*, and *Per2* in the hippocampus were noted at 24–48 h post-injury.^17^ In high-frequency head impact (HFHI) and controlled cortical impact (CCI) mouse models of TBI, dysregulation in the diurnal expression of core circadian genes (*Bmal1*, *Clock*, *Per1*, *Per2*, *Cry1*, and *Cry2*) at 24 h post-TBI was noted.^18^ However, there are no studies to date that have investigated the effect of bTBI on the expression of core circadian clock genes in the hypothalamus and pineal gland at acute or chronic time points or evaluated changes in gene expression patterns of orexin/hypocretin receptors in the hypothalamus during daytime or nighttime.

Our previous work demonstrated that bTBI causes dysregulation of melatonin synthesis and secretion along with changes in melatonin receptors in the pineal gland.^19^ Although circulating levels of melatonin and the stress response hormone, corticosterone, have an inverse relationship, no previous studies evaluated the daytime and nighttime levels of corticosterone after blast exposure. Hence, the goals of the present study were to: 1) investigate the differential expression of core clock genes in the hypothalamus and pineal gland; 2) determine the changes in orexin/hypocretin receptor genes in the hypothalamus; and 3) estimate the levels of corticosterone hormone in the plasma at acute and chronic time points post-blast exposure.

## Methods

### Animals

All animal procedures were performed at the Association for Assessment and Accreditation of Laboratory Animal Care (AAALAC)-accredited Walter Reed Army Institute of Research (Silver Spring, MD) under an Institutional Animal Care and Use Committee (IACUC)-approved protocol. Male 7- to 8-week-old Sprague-Dawley rats (weighing 250–275 g) were obtained from Charles River Laboratories (Wilmington, MA). Rats were housed in individually ventilated cages in a dedicated rodent room maintained at 20°C–22°C on a 12-h light/12-h dark cycle with free access to standard rat chow diet (Prolab IsoPro RMH3000; LabDiet, St. Louis, MO) and chlorinated water *ad libitum*. Lights were turned off at 6:00 pm and turned back on at 6:00 am. Rats were randomly placed into two groups—sham and repeated blast (BB)—with each group containing 6 rats for each time point.

### Blast exposure

Rats subjected to blast exposure were anesthetized with isoflurane (4%) for 6–8 min. Anesthetized rats were secured in a longitudinal prone orientation (rats facing the oncoming blast wave) in an advanced blast simulator. To induce moderate bTBI, a peak positive static pressure of ∼19 psi with a positive phase duration of 4–5 ms was utilized. Rats subjected to tightly coupled repeated blasts were exposed to two blast overpressure waves (∼19 psi) separated by 2 min, as described in our previous study.^20^ Rats in the sham group were anesthetized and placed in the recovery cage without blast exposure.

### Sample collection

Rats were anesthetized with isoflurane and euthanized at 24 h or 1 month after blast injury. For nighttime sampling, samples were collected at 10:00 pm, and for light-phase sampling, samples were collected during daytime at 10:00 am. For samples collected at night, rats were anesthetized in the dark with the aid of night vision lighting (red) and their eyes were shielded from light before tissue collection. The hypothalamus and pineal gland were dissected and stored at −80°C until further analysis. Blood samples were collected in BD vacutainer ethylenediaminetetraacetic acid tubes purchased from Becton, Dickinson and Company (Franklin Lakes, NJ). Plasma was separated by centrifugation at 1000*g* for 15 min, aliquoted, and stored at −80°C until analysis.

### RNA extraction and quantitative real-time polymerase chain reaction

Total RNA was extracted from the rat hypothalamus and pineal gland using the Qiagen RNeasy mini kit (catalog no.: 74104), as per the manufacturer's protocol, and were quantified using a NanoDrop 2000 (ThermoFisherScientific, Waltham, MA). RNA samples with absorbance ratios (260/280 nm) of 1.8–2.0 were considered pure and were transcribed into complementary DNA (cDNA) using an RT^2^ First Strand Kit (catalog no.: 330404; Qiagen, Germantown, MD). Until use, cDNA samples were stored at −20°C. Clock gene primers were obtained from Integrated DNA Technologies (IDT; Coralville, IA), and hypocretin/hypocretin receptor genes were obtained from Qiagen (Table 1). All samples were run in triplicate. Briefly, quantitative real-time polymerase chain reaction (qRT-PCR) reactions were performed using RT^2^ SYBR Green qPCR Mastermix reagent (Qiagen) on an Applied QuantStudio 6 Flex qPCR system (Life Technologies, Grand Island, NY). No template control samples served as negative controls. Differential gene expression was calculated using the 2^−ΔΔCt^ method (genes of interest normalized to β-actin). The data are shown as fold changes in comparison to control groups.

**Table 1.** List of Primer Sequences

Gene	Company	Forward (5’-3’)	Reverse (5’-3’)
*Bmal1*	IDT	GGC TGT TCA GCA CAT GAA AAC	GCT GCC CTG AGA ATT AGG TGT T
*Clock*	IDT	CTT CCT GGT AAC GCG AGA AAG	GTC GAA TCT CAC TAG CAT CTG AC
*Per1*	IDT	GAT GTG GGT GTC TTC TAT GGC	AGG ACC TCC TCT GAT TCG GC
*Per2*	IDT	CAG GTT GAG GGC ATT ACC TCC	AGG CGT CCT TCT TAC AGT GAA
*Cry1*	IDT	CAC TGG TTC CGA AAG GGA CTC	CTG AAG CAA AAA TCG CCA CCT
*Cry2*	IDT	CAC TGG TTC CGC AAA GGA CTA	CCA CGG GTC GAG GAT GTA GA
*Actin*	IDT	GGC CAA CCG TGA AAA GAT GAC C	AAC CCT CAT AGA TGG GCA CAG

Gene	Company	RefSeq accession no.	Catalog no.
*Hcrt*	Qiagen	NM_013179	PPR43894B
*Hcrtr1*	Qiagen	NM_013064	PPR52713A
*Hcrtr2*	Qiagen	NM_013074	PPR44541A

*Hcrt*, hypocretin; *Hcrtr1*, hypocretin receptor 1; *Hcrtr2*, hypocretin receptor 2.

### Corticosterone assay

Quantitative measurements of corticosterone in the plasma were performed using the Detect X corticosterone enzyme-linked immunosorbent assay kit from Arbor Assays (catalog no.: K014-H5; Arbor Assays, Ann Arbor, MI), as per the manufacturer's protocol.

### Statistical analyses

All data are presented as the means ± standard error of the mean (SEM), and differences between the two groups were compared using a two-tailed *t*-test. Differences among groups were considered statistically significant at *p* values <0.05. Statistical analyses were conducted using GraphPad Prism software (version 9; GraphPad Software Inc., La Jolla, CA).

## Results

### Molecular diurnal rhythms of core circadian clock genes in the hypothalamus and pineal gland of non-blasted rats

Previous studies have demonstrated that all the core circadian clock genes express significant circadian rhythms in messenger RNA (mRNA) levels in the SCN of the hypothalamus.^21,22^ These core clock genes have also been found to be rhythmically expressed in other regions of the brain, indicating that extra-SCN core clock gene expression could contribute to neuronal homeostasis.^23,24^ For instance, the SCN is considered as a melatonin rhythm generator as bilateral SCN lesions lead to the abolishment of circadian melatonin synthesis and secretion in the pineal gland.^25^ Only limited studies have been performed to determine the nature of all the core circadian clock genes in the pineal gland and whether there is a regulatory loop that exists between the SCN and pineal gland. Hence, we investigated the diurnal rhythm of gene expression of core circadian clock genes in the hypothalamus and pineal gland at two different time points (during the day and at night) in non-blasted (sham control) rats.

**FIG. 1.**
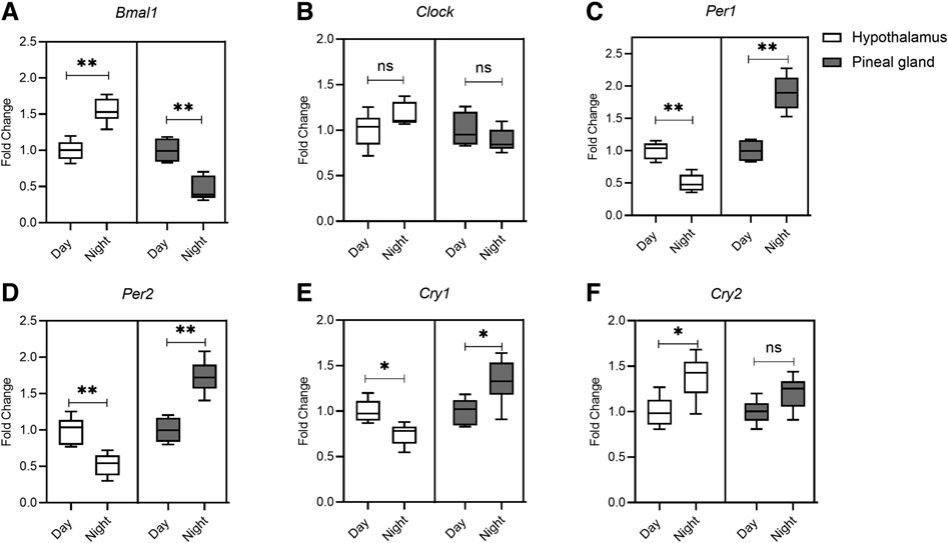
Circadian core clock gene expression changes during the day and at night in the hypothalamus (green) and pineal gland (red). Fold changes in expressions were quantified for (**A**) *Bmal1*, (**B**) *Clock*, (**C**) *Per1*, (**D**) *Per2*, (**E**) *Cry1*, and (**F**) *Cry2* mRNA. Results are expressed as means ± SEM (*n* = 6); **p < 0.01 and *p < 0.05 for sham versus blast group. mRNA, messenger RNA; ns, not significant; SEM, standard error of the mean.

Expression of all the core circadian clock genes, except the *clock* gene (Fig. 1B), were found to follow a diurnal rhythm in the hypothalamus. *Bmal1* and *Cry2* expressions were higher during the night (Fig. 1A,E), whereas the expression levels of *Per1*, *Per2*, and *Cry1* (Fig. 1C–E) were higher during the daytime. Interestingly, in the pineal gland, we have observed a statistically significant decrease in *Bmal1* expression (Fig. 1A), along with increased expression levels of *Per1*, *Per2*, and *Cry1* at nighttime (Fig. 1C–E).

### Blast-induced traumatic brain injury induces alterations in transcript levels of core clock genes in the pineal gland

**FIG. 2.**
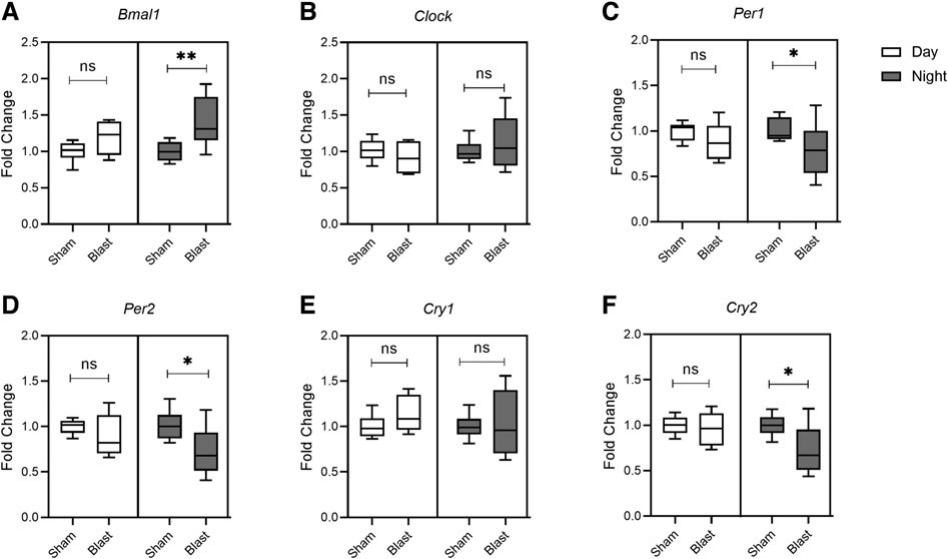
Blast exposure alters expression of core circadian clock genes in the pineal gland during daytime (green) and at nighttime (red). Fold change expressions were quantified for (**A**) *Bmal1*, (**B**) *Clock*, (**C**) *Per1*, (**D**) *Per2*, (**E**) *Cry1,* and (**F**) *Cry2* mRNA. Results are expressed as means ± SEM (*n* = 6); **p < 0.01 and *p < 0.05 for sham versus blast group. mRNA, messenger RNA; ns, not significant; SEM, standard error of the mean.

We investigated transcripts of core circadian clock genes (*Bmal1*, *clock*, *Per1*, *Per2*, *Cry1*, and *Cry2*) in the pineal gland samples collected during the daytime and nighttime, at acute (24 h) and chronic (28 days) time points after bTBI. In samples collected at night after 24 h post-bTBI, *Bmal1* (*p* = 0.004) was significantly increased (Fig. 2A), whereas expression levels of *Per1* (*p* = 0.04), *Per2* (*p* = 0.024), and *Cry2* (*p* = 0.04) were significantly decreased in comparison to the sham group (Fig. 2C,D,E). Transcript levels of *Clock* (*p* = 0.73; Fig. 2B) and *Cry1* (*p* = 0.86) genes (Fig. 2E) were unchanged in the pineal gland samples collected during night at 24 h post-bTBI. All six transcripts did not show statistically significant changes in the pineal gland samples collected during the daytime at 24 h post-bTBI, though there was a similar trend of an increase in *Bmal1* and a decrease in *Per1*, *Per2*, and *Cry2* (Fig. 2A–E). At 28 days post-bTBI, there was no statistically significant difference in expression levels of any of the six core circadian clock genes between the bTBI and sham groups, in samples collected during the daytime or nighttime. The results with the statistical significance (*p* value) are summarized in Table 2.

**Table 2.** Fold Change mRNA Expression of Clock Genes, Hypocretin, and Hypocretin Receptors at 1 Month Post-Blast Exposure

Gene	Pineal gland (night samples)		Pineal gland (day samples)		Hypothalamus (night samples)		Hypothalamus (day samples)	
	Fold change	*p *value	Fold change	*p *value	Fold change	*p *value	Fold change	*p *value
*Bmal1*	1.05	0.69	0.95	0.87	1.06	0.29	1.09	0.15
*Clock*	1.14	0.39	1.05	0.39	0.78	0.22	0.92	0.70
*Per1*	1.27	0.09	1.12	0.18	0.97	0.84	1.06	0.88
*Per2*	1.12	0.06	1.09	0.12	0.90	0.69	1.01	0.44
*Cry1*	1.16	0.31	1.18	0.47	0.87	0.09	0.93	0.21
*Cry2*	1.04	0.42	0.87	0.14	1.07	0.84	1.02	0.53
*Hcrt*	N/A	N/A	N/A	N/A	0.88	0.40	0.80	0.34
*Hcrtr1*	N/A	N/A	N/A	N/A	1.20	0.11	1.30	0.34
*Hcrtr2*	N/A	N/A	N/A	N/A	1.10	0.70	1.21	0.20

mRNA, messenger RNA; *Hcrt*, hypocretin; HCRTR1, hypocretin receptor 1; HCRTR2, hypocretin receptor 2; N/A, not applicable.

### Blast-induced traumatic brain injury induces alterations in transcript levels of core clock genes in the hypothalamus

**FIG. 3.**
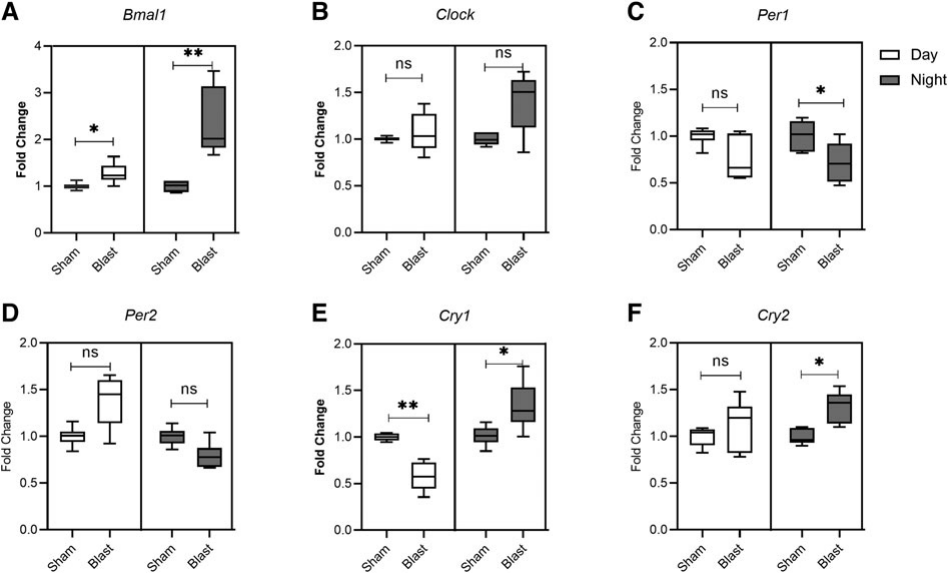
Blast exposure alters expression of core circadian clock genes in the hypothalamus during daytime (green) and at nighttime (red). Fold changes in expressions were quantified for (**A**) *Bmal1*, (**B**) *Clock*, (**C**) *Per1*, (**D**) *Per2*, (**E**) *Cry1*, and (**F**) *Cry2* mRNA. Results are expressed as means ± SEM (n = 6); **p < 0.01 and *p < 0.05 for sham versus blast group. mRNA, messenger RNA; ns, not significant; SEM, standard error of the mean.

Next, changes in the transcripts of core circadian clock genes (*Bmal1*, *Clock*, *Per1*, *Per2*, *Cry1*, and *Cry2*) in the hypothalamus samples collected during the daytime and nighttime, at acute (24 h) and chronic (28 days) time points after bTBI, were investigated. In samples collected at night, 24 h post-bTBI, *Bmal1* (*p* = 0.002; Fig. 3A), *Cry1* (*p* = 0.03; Fig. 3E), and *Cry2* (*p* = 0.015; Fig. 3F) were significantly increased. Transcript levels of *Per1* (*p* = 0.026; Fig. 3C) was decreased. Expression levels of *Clock* showed a non-statistically significant increase (*p* = 0.06; Fig. 3B) whereas *Per2* showed a non-statistically significant decrease (*p* = 0.15; Fig. 3D) in the samples collected at night, 24 h post-bTBI.

In the samples collected during the daytime at 24 h post-injury, only *Bmal1* showed a statistically significant increase in the blast group (*p* = 0.01; Fig. 3A), whereas *Cry1* decreased (*p* = 0.002; Fig. 3E). All the other transcripts—*Clock* (*p* = 0.48; Fig. 3B), *Per1* (*p* = 0.09; Fig. 3C), *Per2* (*p* = 0.06; Fig. 3D), and *Cry2* (*p* = 0.31; Fig. 3F)—did not show statistically significant changes in the hypothalamus samples collected during the daytime at 24 h post-bTBI. At 28 days post-bTBI, no statistically significant differences in any of the six core clock genes in samples collected during the daytime or nighttime were noted. The results with the statistical significance (*p* value) are summarized in Table 2.

### Blast-induced traumatic brain injury induces alterations in messenger RNA levels of hypocrytin and its receptors in the hypothalamus

Several lines of evidence indicate that the hypothalamic neuropeptides, such as hypocretins (also identified as orexin), play a key role in the regulation of sleep-wake behavior. Furthermore, hypocretin and its receptor subtypes hypocretin receptor 1 (HCRTR1) and hypocretin receptor 2 (HCRTR2) exhibit diurnal rhythmicity, and increased activity of hypocretin neurons are noted during the wake phase and correlate with motor activity in nocturnal rodents.^13,14^ Additionally, mRNA levels of hypocretin receptors significantly correlate with the diurnal rhythmicity of clock genes. In particular, the expression pattern of *Hcrtr2* shows a strong correlation with that of *Bmal1* in the hypothalamus. Because the rhythmic expression of hypocretin and its receptors is linked to clock gene expression and hypocretin signaling plays a key role in the timing of sleep-wake behavior, we sought to investigate the effect of bTBI on *Hcrt* and its receptor subtypes (*Hcrtr1* and *Hcrtr2*) in hypothalamus samples at acute (24 h) and chronic (28 days) time points after blast injury. The effects of bTBI were analyzed separately for the light and dark phases.

**FIG. 4.**
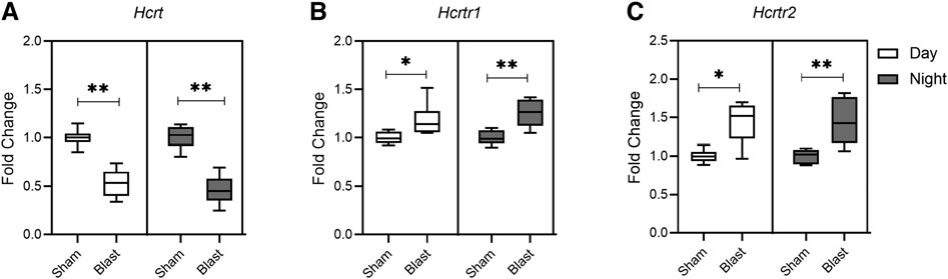
Blast exposure alters expression of hypocretin (*Hcrt*) and hypocretin receptors (*Hcrtr1* and *Hcrtr2*) in the hypothalamus during daytime (green) and at nighttime (red). Fold changes in expressions were quantified for (**A**) *Hcrt*, (**B**) *Hcrtr1*, and (**C**) *Hcrtr2* mRNAs. Results are expressed as means ± SEM (*n* = 6); **p < 0.01 and *p < 0.05 for sham versus blast group. mRNA, messenger RNA; SEM, standard error of the mean.

At 24 h post-bTBI, we observed a statistically significant decrease in mRNA levels of *Hcrt* (*p* = 0.002; Fig. 4A) and an increase in *Hcrtr1* (*p* = 0.008; Fig. 4B) and *Hcrtr2* expression (*p* = 0.009; Fig. 4C) in the blast group compared to the sham group in samples collected during the nighttime. Similarly, we observed a corresponding decrease in *Hcrt* (*p* = 0.002; Fig. 4A) and an increase in *Hcrtr1* (*p* = 0.0152; Fig. 4B) and *Hcrtr2* expression (*p* = 0.026; Fig. 2C) in the blast group when compared to sham in samples collected during the daytime. At 28 days post-bTBI, we did not see any statistically significant changes in mRNA levels of *Hcrt*, *Hcrtr1*, or *Hcrtr2* in the blast group when compared to the sham group. The results with the statistical significance (*p* value) are summarized in Table 2.

### Blast-induced traumatic brain injury induces alterations in plasma corticosterone levels

The SCN plays a crucial role in regulating daily rhythms in hormone production and these rhythms are lost after SCN ablation.^26^ Specifically, the diurnal rhythm of corticosterone secretion is controlled by the SCN,^27–29^ and the circadian peak of corticosterone release occurs during the active phase of the animal. In diurnal animals, the peak release is noted during the early morning, and in nocturnal animals such as rats, the plasma corticosterone levels rise during the early night.^30–33^ Further, daily corticosterone release is critically regulated by coordinated clock gene expression in the SCN and paraventricular nucleus.^34^ Hence, changes in core clock gene expression could alter corticosterone synthesis and release. Thus, we investigated the effect of blast exposure on plasma corticosterone levels at 24 h and 28 days post-blast injury.

**FIG. 5.**
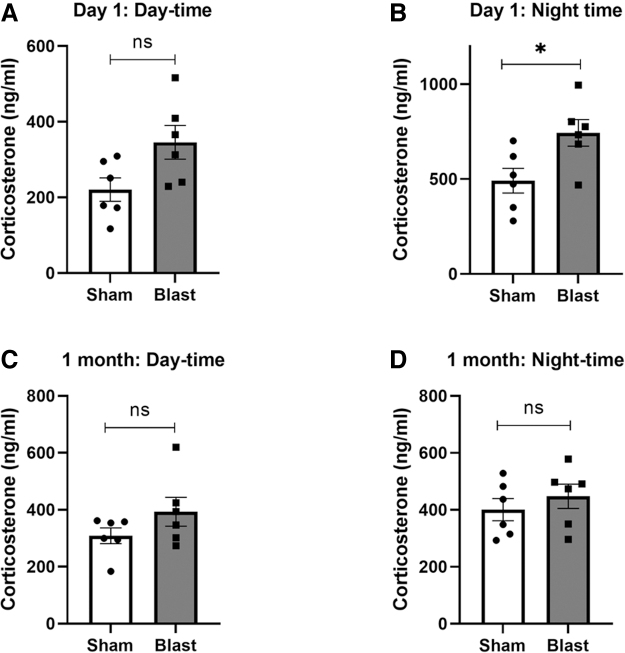
Plasma corticosterone levels measured at (**A**) 24 h post-injury during daytime; (**B**) 24 h post-injury during nighttime; (**C**) 1 month post-injury during daytime; and (**D**) 1 month post-injury during nighttime. Results are expressed as means ± SEM (*n* = 6); **p < 0.01 and *p < 0.05 for sham versus blast group. mRNA, messenger RNA; ns, not significant; SEM, standard error of the mean.

Our results indicated that plasma corticosterone levels were increased in blast-exposed rats at 24 h, and the increase was more pronounced in samples collected during the nighttime (*p* = 0.041; Fig. 5A). Though there was an increase in plasma corticosterone levels at 24 h post-blast in the samples collected during the daytime, it was not statistically significant (*p* = 0.065; Fig. 5B). We did not observe any significant changes in plasma corticosterone levels in blast-exposed rats at 28 days, both in samples collected during the daytime (*p* = 0.31; Fig. 5C) and nighttime (*p* = 0.24; Fig. 5D).

## Discussion

Previous studies have demonstrated that bTBI patients suffer from acute and chronic sleep disturbances, such as insomnia and anxiety symptoms,^35–37^ indicating a potential disruption to circadian rhythms post-bTBI. Among these, studies that have evaluated the molecular signaling mechanisms involved in the altered sleep-wake cycle and circadian sleep disturbances post-bTBI are very limited. A few pre-clinical studies on non-blast TBI models have shown dysregulation of the circadian clock genes in the SCN and other regions of the brain that are involved in the disruption of circadian rhythms.^17,18^ However, to date, no studies have determined the differential expressions of circadian clock genes in the hypothalamus or pineal gland at acute and chronic time points post-bTBI. To our knowledge, this is the first study to demonstrate changes in the diurnal expression of core circadian clock genes post-bTBI in the hypothalamus and pineal gland of rats. We also report changes in the diurnal expressions of hypocretin and its receptors and associated plasma corticosterone levels after blast injury.

Circadian rhythm is mainly controlled by the SCN, although multiple circadian pacemakers exist in different regions of the brain and in the periphery.^38,39^ We have evaluated the diurnal circadian expression of clock genes in the hypothalamus and pineal gland of uninjured sham-treated rats and found that all six core clock genes show circadian rhythms, although the fold change values vary among genes. Interestingly, the *Bmal1* expression pattern in the hypothalamus and pineal gland was opposite to the *Per1, Per2*, and *Cry1*, maybe because *Bmal1* and *Clock* represent positive loops whereas *Per* and *Cry* genes represent negative loops of transcriptional feedback influencing circadian rhythm.^40^ No significant diurnal changes were noted in the *clock* gene, whereas *Cry2* was increased at night in the hypothalamus and pineal gland. These results are similar to other studies that have evaluated changes in core circadian clock genes in the SCN and pineal gland in rodents and other animal models.^41–43^

We found that bTBI significantly altered the expression of core clock genes in the hypothalamus and pineal gland, with predominant effects noted during the nighttime. In the pineal gland, we have noted an increase in *Bmal1* with associated decreases in *Per1*, *Per2*, and *Cry2* in samples collected at night after 24 h of blast injury. Similarly, in the hypothalamus, increases in *Bmal1* and *Clock* with decreases in *Per1* and *Per2* were noted. Interestingly, *Cry1* and *Cry2* expressions were increased in the hypothalamus of blast-injured rats. In a lateral fluid-percussion model of TBI, increased expression of *Cry1* mRNA at 20 h after injury and *Bmal1* mRNA at 44 h after injury in the SCN were noted.^17^ Similar expression changes in core clock genes in the SCN were noted in HFHI and CCI models after 24 h of injury.^18^ Our results are in line with other studies that demonstrated *Bmal1* mRNA acrophase (peak) at Zeitgeber time (ZT) 18 in the dark phase with *Per1* and *Per2* antiphase to *Bmal1* mRNA in the SCN. The acrophase of *Per1* mRNA occurred at ZT5, whereas the acrophase of *Per2* occurred at ZT9.^21,44^ Similarly, *Bmal1* expression in the pineal gland peaked at ZT6 and reached trough levels at ZT18, whereas *Per1* and *Per2* mRNA expression peaked at ZT16 and ZT20, respectively, which is converse to what is observed in the SCN.^42^ The significance of this opposite peak and trough mRNA expression of core circadian clock genes in the SCN and pineal gland needs further exploration.

The orexinergic/hypocretinergic system plays a vital role in promoting arousal states along with other biological functions, such as food and fluid intake, pain, memory, and glucose metabolism.^45,46^ Blast injury downregulated the expression of *Hcrt* gene along with triggering an increased expression of *Hcrtr1* and *Hcrtr2* at 24 h post-injury during both the daytime and nighttime. Our results are in line with previous studies that have demonstrated that within 3 days of insult, TBI reduced hypocretin in the hypothalamus and blunted diurnal rhythm of hypocretin between the light and dark phases.^47^ At 1 month after blast injury, although the changes were similar to those observed at 24 h, they were not statistically significant. Previous studies have reported a decrease in hypocretin-producing cells in the hypothalamus post-TBI even at chronic time points extending to 15 days post-injury and later.^48,49^ Further, in TBI patients, orexin-A levels in the cerebrospinal fluid were lowered during the acute phase and persisted up to 6 months post-TBI.^50,51^ In contrast, no changes in orexin-A–positive cells were observed at 24 or 72 h post-blast in the hypothalamus of mice exposed to ∼15 psi blast overpressure waves.^5**2**^ No previous studies have evaluated hypocretin receptor expressions in the hypothalamus post-bTBI. One study, in a mouse model of intracerebral hemorrhage, showed decreased protein expression of OXA (*Hcrt*) and increased OXR1 (*Hcrtr1*) and OXR2 (*Hcrtr2*) at 3 h and continuing up to 72 h post-injury.^53^ Our results, along with previous reports, indicate that TBI, including bTBI, can cause dysregulation of the orexin/hypocretin system, which can affect the sleep-wake cycle, mandating further investigation.

Cortisol is a stress hormone associated with stress response. In patients with severe TBI, cortisol levels are elevated within a few hours post-injury and gradually decrease during patient recovery, indicating that the dynamic changes in cortisol levels may serve as a prognostic biomarker of TBI.^54^ The daily release of cortisol, or corticosterone in rats, depends on the coordinated clock gene and neuronal activity rhythms in hypothalamic neurons, specifically SCN and paraventricular neurons.^34^ Hence, injury-related dysfunction in the hypothalamus could affect corticosterone release. Blast injury caused an increase in plasma corticosterone levels at 24 h post-injury. The changes were more prominent in samples collected at night, the time during which corticosterone levels were reported to reach the peak.^30–33^ At 1 month post-blast injury, plasma corticosterone levels were near normal. In animal models of mild-to-moderate TBI, elevated resting plasma corticosterone levels at 6 and 24 h post-injury were similarly noted,^55,56^ followed by a decrease at 2 months post-injury.^57^

Our results, along with other previous reports, indicate that corticosterone levels are elevated in the acute period post-TBI and return to normal levels at 1 month. Understanding the factors promoting an acute rise in corticosterone along with acute changes in the circadian clock genes, particularly in the hypothalamus post-bTBI, may provide insight into the molecular underpinnings to secondary injuries and elucidate novel treatment strategies.

Thus, we have identified circadian clock gene expression changes during the day and night at acute and chronic time points post-blast. Importantly, we found that several clock genes exhibited diurnal changes in expression patterns, mainly in the hypothalamus, after blast injury. In addition, we found changes in orexin/hypocretin and its receptor expression changes in the hypothalamus after blast injury. We postulate that changes in the clock genes, hypocretin and its receptors, after blast exposure cause alterations in circadian rhythmicity that might contribute to sleeping difficulties and functional recovery post-bTBI. In order to further test this hypothesis, a follow-up study conducted in a diurnal animal model of bTBI is warranted.

## Transparency, Rigor, and Reproducibility Summary

All rats used were grouped randomly and housed 2 rats per cage. Each group had 6 rats, and the rats from each shipment were distributed uniformly into each of the three groups to reduce batch-to-batch variations from the vendor. The sample size of 6 was selected based on power calculation, and the selected sample size gave >80% power. No mortality was observed after repeated blast exposures. Investigators who participated in the study were blinded to the experimental groups. Blast pressure was measured for each exposure to confirm that rats were exposed to comparable overpressure waves. Samples from all rats were collected and stored at −80°C until analysis. Analysis of samples was carried out in a single batch to avoid variability between different batches of analyses. Data from this study will be available upon formal request to the corresponding author.

## Abbreviations Used

AAALAC: Association for Assessment and Accreditation of Laboratory Animal Care
bTBI: blast-induced TBI
CCI: controlled cortical impact
cDNA: complementary DNA
*Hcrt*: hypocretin
HCRTR1: hypocretin receptor 1
HCRTR2: hypocretin receptor 2
HFHI: high-frequency head impact
IACUC: Institutional Animal Care and Use Committee
mRNA: messenger RNA
qRT-PCR: quantitative real-time polymerase chain reaction
SCN: suprachiasmatic nucleus
TBI: traumatic brain injury
ZT: Zeitgeber time
